# An Online Prescription for Hospitalisation: Euglycaemic Ketoacidosis Caused by an Online Tirzepatide Prescription

**DOI:** 10.7759/cureus.97410

**Published:** 2025-11-21

**Authors:** Thibagaran Sathambihai, Kushagra Mathur, Seshnag Siddavaram

**Affiliations:** 1 General Medicine, Guy's and St Thomas' NHS Foundation Trust, London, GBR; 2 General Internal Medicine, Medway NHS Foundation Trust, Gravesend, GBR; 3 Acute Internal Medicine, Dartford and Gravesham NHS Trust, Dartford, GBR

**Keywords:** euglycaemic ketoacidosis, metabolic acidosis, online prescribing, starvation, tirzepatide

## Abstract

Obesity affects a significant proportion of the UK population, and tirzepatide, a dual glucagon-like peptide (GLP-1) and gastric inhibitory polypeptide (GIP) receptor agonist, is increasingly being prescribed by online services as well as primary care for weight loss in both diabetic and non-diabetic patients. We present the case of a 61-year-old woman with no past medical history, who developed euglycaemic ketoacidosis following six weeks of tirzepatide (Mounjaro®) therapy, which was initiated via an online consultation for weight loss. Despite normal blood glucose levels, she exhibited significant ketonaemia and metabolic acidosis, which resolved with intravenous fluid resuscitation and replacement. This case brings to light the importance of treating clinicians being aware of the potential adverse side effects of euglycaemic ketoacidosis in patients using tirzepatide, and highlights the risk when these medications are prescribed via novel care pathways, which have less rigid monitoring paradigms than traditional prescribing models. Early recognition of, and prompt management, are important to ensure the mitigation of morbidity associated with this under-recognised adverse effect.

## Introduction

Obesity is a significant and growing medical issue; 27% of the population of the United Kingdom was said to be obese in 2023 [1]. Non-pharmacological approaches, such as diet and exercise, are first-line management strategies for obesity, but novel drugs like glucagon-like peptide (GLP-1) and gastric inhibitory polypeptide (GIP) receptor agonists show promising results for weight loss [2].

Currently, tirzepatide is recommended by the National Institute for Health and Care Excellence (NICE) for weight loss, alongside a reduced-calorie diet and increased physical activity. Current recommendations for tirzepatide therapy are for patients who have a BMI >35 kg/m² and at least one weight-related comorbidity, such as hypertension, type 2 diabetes, dyslipidaemia, obstructive sleep apnoea, or cardiovascular disease. NICE guidance also requires the drug manufacturer to agree to provide tirzepatide according to the commercial arrangement [3].

The mechanism of action of tirzepatide is well understood, combining both GLP-1 and GIP receptor agonism. GLP-1 receptor agonism stimulates insulin secretion from pancreatic beta cells in a glucose-dependent manner and suppresses glucagon release from alpha cells during hyperglycaemia. GLP-1 agonism also delays gastric emptying, reducing postprandial sugar spikes, as well as suppressing appetite to further reduce caloric intake [4].

GIP receptor agonism increases insulin release more effectively than GLP-1 agonism alone, and, unlike GLP-1 alone, can increase glucagon secretion during episodes of hypoglycaemia. Combination formulations offer the potential to increase weight loss beyond the effects of GLP-1 agonists alone.

As tirzepatide therapy increases in popularity, it is important for healthcare professionals to be aware of the adverse effects that may occur during the initial lead-in period of treatment. The SURMOUNT-4 study, which looked at the ongoing weight loss achieved by patients who continued tirzepatide therapy for 52 weeks after an initial treatment lead-in period, found that 81% of study participants experienced at least one adverse effect during the initial 36 weeks of therapy. The adverse effects were most commonly gastrointestinal: vomiting, nausea, diarrhoea, and abdominal pain. Study participants who continued tirzepatide therapy reported a reduction in the severity of adverse effects, and the gastrointestinal side effects were reported to be mild to moderate [5]. In this case report, we describe a significant adverse effect that occurred early in the treatment period and required the patient to be hospitalised. 

## Case presentation

A 61-year-old office worker with a BMI of 31 kg/m², with no significant past medical history and no regular medications, presented to the Emergency Department with abdominal pain. Six weeks prior to admission, due to her anxiety about her weight and the increased media attention surrounding weight-loss prescriptions, she sought a consultation with an online prescription service and was prescribed subcutaneous tirzepatide for weight loss after an online consultation. Tirzepatide 5 mg subcutaneously was given every week for six weeks prior to the development of her complication, which required hospital admission. 

She presented to the Emergency Department with intermittent loose stools for four weeks, but no diarrhoea, decreased appetite, or vomiting for two weeks. 

After initial examination and assessment, she was found to be clinically dehydrated, with unremarkable inflammatory markers. Her capillary blood ketones on presentation were 5.4 mmol/L. A venous blood gas on presentation demonstrated a high anion gap metabolic acidosis, and a repeat gas after fluid resuscitation and replacement demonstrated improvement and resolution of her acidosis (Table 1). 

**Table 1:** Venous blood gas demonstration resolution of acidosis FiO_2_ interpretation is not routinely required for venous blood gas interpretation.

Venous Blood Gas	Admission	After Treatment	Reference Range
FiO_2_ (%)	21%	21%	-
pH	7.25	7.3	7.3 - 7.4
pCO_2_ (kPa)	3.9	3.5	4.3 - 6.4
pO_2_ (kPa)	3.4	5	11.0 - 14.4
HCO_3_⁻ (mmol/L)	12.7	14.7	22 - 28
Lactate (mmol/L)	1.6	1.4	0.4 - 0.8
Potassium (mmol/L)	3.9	3.4	3.5 - 5.1
Sodium (mmol/L)	138	140	135 - 145
Glucose (mmol/L)	6.2	5.4	3.6 - 5.3

A thorough history and examination, including family history, was elicited, and, after considering her laboratory findings, a diagnosis of drug-induced euglycaemic ketoacidosis was made. She was admitted to a medical ward to continue intravenous fluid resuscitation with 0.9% sodium chloride at 250 mL per hour, and she clinically improved over the next eight hours. On discharge, her capillary blood ketones were <0.2 mmol/L. She was discharged home with advice to stop the causative medication. 

## Discussion

Ketoacidosis, though uncommon, is a serious adverse effect with this drug [6]. The mechanism by which ketoacids are produced is well understood, and, in this case, it is likely that the gastrointestinal side effects experienced during the treatment initiation period contributed to a state of starvation. Ketoacids are produced when adipose tissue breaks down triglycerides into glycerol and free fatty acids in response to low insulin and high glucagon levels. In the liver, free fatty acids are converted to acetyl-CoA, which produces NADH and FADH_2_ for energy ​[7]. 

When insulin is scarce in the body, the excess acetyl-CoA forms ketone bodies, which provide an alternate energy source for the brain and muscles. The accumulation of these ketoacids leads to metabolic acidosis. Ketoacidosis is a dangerous physiological state, and the correction of the underlying driver of the acidosis is critical. Normal blood glucose levels can be a confounding factor when trying to establish the mechanism of ketoacidosis ​[8]. 

In the context of this case report, the patient’s blood glucose level was normal, but the ketone levels rose significantly due to a combination of dual GLP-1/GIP agonists and gastrointestinal side effects, such as nausea, vomiting, and abdominal pain, contributing to a state of starvation. 

Other case reports, such as those from Louwagie et al. [9] and Iqbal et al. [10], have recently been published and contribute to the emerging awareness of the effects of tirzepatide therapy, particularly emphasising the risk of morbidity associated with developing ketoacidosis. 

Louwagie et al. report a case of euglycaemic ketoacidosis in the context of co-administration of sodium-glucose co-transporter 2 (SGLT2) inhibitors, where the physiological derangement, namely the acidosis, was so severe that the patient required admission to an intensive care unit (ICU) for hemofiltration [9]. This case particularly emphasises the risk of developing acidaemia when SGLT2 inhibitors are prescribed with tirzepatide. 

Iqbal et al. also report a case of tirzepatide administration causing ketoacidosis. The tirzepatide in this instance was procured over the counter without physician oversight, and this report again described a ketoacidosis so severe that the patient required admission to the ICU. Iqbal et al. suggest the patient’s adverse effect was compounded by underlying insulin resistance [10]. This case further underlines the risk tirzepatide poses when initiated outside of traditional prescribing pathways. 

We advise that healthcare professionals should be aware of this adverse effect in patients presenting with gastrointestinal symptoms and unexplained ketoacidosis. Our case particularly highlights the risk of treatment initiation in patients who have no reported medical history and who have sought pharmacological therapy online. This adverse effect of tirzepatide therapy is particularly important to be aware of, as novel treatment pathways become more popular and increasing numbers of patients are prescribed medications outside conventional treatment systems. 

## Conclusions

Euglycaemic ketoacidosis is a rare but significant complication of tirzepatide therapy in non-diabetic patients. This case highlights the risks of online prescribing and the importance of patient education and monitoring when initiating novel medications. Metabolic acidosis with elevated ketones can occur despite normal glucose levels. Prompt fluid resuscitation and discontinuation of tirzepatide led to full clinical recovery. Clinicians should be aware of starvation-induced ketoacidosis in patients using dual GLP-1 and GIP receptor agonists for weight loss, particularly when patients with no medical history are prescribed tirzepatide through novel prescribing pathways. 
